# Recognition of and care-seeking for maternal and newborn complications in Jayawijaya district, Papua province, Indonesia: a qualitative study

**DOI:** 10.1186/s41043-017-0122-0

**Published:** 2017-12-21

**Authors:** Alfonso Rosales, Sigit Sulistyo, Oktarinda Miko, Lila K. Hairani, Meita Ilyana, Joanne Thomas, Emily Hirata, Rhonda Holloway, Michael Bantung, Kristina Pabate, Candra Wijaya, Dennis Cherian

**Affiliations:** 1 Department of Health, World Vision US, 300 I Street NE, Washington, DC 20002 USA; 2 World Vision Indonesia, Jl. Wahid Hasyim 33, Jakarta Pusat, DKI, Jakarta, Indonesia; 3 Center of Epidemiology Research and Surveillance, University of Indonesia (CERS UI), Faculty of Public Health, University of Indonesia, Building G, 2nd Floor, Room 201, UI Campus, Depok, West Java 16424 Indonesia

**Keywords:** Maternal and newborn mortality, Indonesia, Qualitative study, Illness recognition, Care-seeking, Maternal health, Newborn health

## Abstract

**Background:**

Indonesia’s progress on reducing maternal and newborn mortality rates has slowed in recent years, predominantly in rural areas. To reduce maternal and newborn mortality, access to quality and skilled care, particularly at the facility level, is crucial. Yet, accessing such care is often delayed when maternal and newborn complications arise. Using the “Three Delays” model originated by Thaddeus and Maine (1994), investigation into reasons for delaying the decision to seek care, delaying arrival at a health facility, and delaying the receiving of adequate care, may help in establishing more focused interventions to improve maternal and newborn health in this region.

**Methods:**

This qualitative study focused on identifying, analyzing, and describing illness recognition and care-seeking patterns related to maternal and newborn complications in the Jayawijaya district of Papua province, Indonesia. Group interviews were conducted with families and other caregivers from within 15 villages of Jayawijaya who had either experienced a maternal or newborn illness or maternal or newborn death.

**Results:**

For maternal cases, excessive bleeding after delivery was recognized as a danger sign, and the process to decide to seek care was relatively quick. The decision-making process was mostly dominated by the husband. Most care was started at home by birth attendants, but the majority sought care outside of the home within the public health system. For newborn cases, most of the caregivers could not easily recognize newborn danger signs. Parents acted as the main decision-makers for seeking care. Decisions to seek care from a facility, such as the clinic or hospital, were only made when healthcare workers could not handle the case within the home. All newborn deaths were associated with delays in seeking care due to caretaker limitations in danger sign identification, whereas all maternal deaths were associated with delays in receiving appropriate care at facility level.

**Conclusions:**

For maternal health, emphasis needs to be placed on supply side solutions, and for newborn health, emphasis needs to be placed on demand and supply side solutions, probably including community-based interventions. Contextualized information for the design of programs aimed to affect maternal and newborn health is a prerequisite.

## Background

The government of Indonesia has identified reduction of maternal and neonatal mortality as a national priority. The maternal mortality ratio (MMR) in Indonesia was recorded as 359 maternal deaths per 100,000 live births whereas the neonatal mortality rate (NMR) was 19 per 1000 live births during 2008–2012 [1]. Each year, there are more than 60,000 newborn deaths in Indonesia, which account for 48% of under-five deaths in the country. Newborn deaths are 60% higher for those residing in a rural setting [2] and 42% higher than the national average when residing in Papua province [2]. Jayawijaya district, where the study took place, has a newborn mortality 60% higher than the Papua province average [1, 2].

Service provision in Jayawijaya district is provided mostly by community health centers (*puskesmas*) available in every sub-district and a public referral hospital located in the district. The main tasks of *puskesmas* have been basic care services, immunization, antenatal, childbirth services, and postnatal care, sanitation, and dental services. In 2014, the government of Indonesia implemented a National Social Insurance (JKN) program that aims to cover the full population by 2019. The premiums for poor and near-poor citizens are subsidized by the government. In 2015, 87.5% of the Jayawijaya population was covered by health insurance, including 42% of the poor population [3]. Additionally, in 2006, the Ministry of Health launched the community-based health program (UKBM), which includes Alert Village (*Desa Siaga*) and village health posts (*poskesdes*). Every *poskesde* must be served by one midwife and one nurse [1]. In this area of the country, most health service delivery is provided by government facilities. There are currently 14 healthcare facilities in the district (one district hospital and 13 *puskesmas*). The great majority of people in Jayawijaya belong to the Dani ethic group (ethnic and linguistic group living in the mountain region of Jayawijaya district, in the Indonesian province of Papua), many of whom typically have very low coverage of basic health services. According to the WHO Partnership for Maternal, Newborn and Child Health (PMNCH) [4], reductions in newborn mortality require access to quality and skilled care, particularly at the facility level [5]. However, accessing such care is often delayed when newborn complications arise. Evidence on access to care is lacking from Indonesia, and particularly from the Papua province, as to how women and families identify maternal and newborn complications, the factors behind the decision-making process to seek care, and the influential role of cultural beliefs.

Moreover, because Indonesia does not yet have a complete recording of maternal or neonatal deaths through a vital registration system, the number of reported deaths reflects only those reported to the health services and is likely to underestimate population death rates. Although information coming out from hospital records is useful in identifying factors and causes associated with these deaths, it provides little information on determinants of care-seeking on those deaths occurring outside the formal health system. Studies from countries other than Indonesia suggest many barriers to seeking necessary skilled care. Preference for care from traditional providers and home remedies are cited most often [6–20]. Culturally shared beliefs were also a common factor influencing delay of skilled care [6–10, 12, 17, 19–22]. In addition, challenges were apparent in recognizing symptoms that indicate potentially life-threatening complications [7, 12, 18, 19, 21, 23–25]. Other barriers include limited decision-making power among women [3, 10, 12, 16, 22, 23, 26]; changing social norms, such as socioeconomic status or maternal education [7, 18, 20, 21]; inefficient access to care, including inadequate local facilities and lack of rapid transportation [3, 7, 18, 20]; and doubtful perceptions regarding the overall effectiveness and quality of care available from skilled health providers at local health centers [27].

To produce locally relevant information and inform strategies to reduce maternal and newborn mortality, a study on care-seeking behaviors was undertaken in rural Indonesia by World Vision in collaboration with Indonesia’s National Institute of Health Research and Development, Ministry of Health, and the University of Indonesia, with support from United States Agency for International Development’s (USAID) Translating Research into Action (TRAction) project, a multi-country implementation research project.

## Methods

The focus of this study was to identify, analyze, and describe illness recognition and care-seeking patterns related to maternal and newborn complications among Dani people residing in Jayawijaya district, Papua province, Indonesia. Research questions were described in a previous paper [28].

### Study setting

The study was conducted in Jayawijaya district, Papua province in eastern Indonesia, where 82% of the population belongs to the Dani ethnic group. At the time of this research, the district carried a population of 204,112 people, in 32 sub-districts divided into 366 villages [29] with majority of the population being Christian. A high proportion of the community was poor (42%), and approximately 72% of women deliver in the home and 64% deliver with unskilled attendants [30]. This area is isolated with no road links to the provincial capital and only a few roads within each sub-district. The communities are in a state of social transition following the sustained contact with the outside world and the decentralization of funding management at the district and village level. The local government has a policy of free healthcare for the communities, and the national government has implemented social health insurance since January 2015, which provides poor communities free access to healthcare services.

### Data collection

This study involved collecting qualitative data from families and caregivers who have experienced newborn death or severe illness or maternal death or postpartum hemorrhage (PPH). Data sources used to identify and locate cases included hospital records (district public hospital), health center (*puskesmas*) records, midwives’ (*poskesdes*) records, community health worker (CHW) records, community-based leaders (religious and others), and community-based screenings. For maternal cases, 15 individuals were interviewed (four PPH survival women, six husbands, four close relatives, and one health provider); for newborn cases, 21 individuals were interviewed (ten mothers, six fathers, four close relatives, and one health worker). To identify and select cases, the research team focused on case identification from within an area of 5 km around the city of Wamena. After cases were identified, illness narratives were taken. These illness narratives consisted of stories and interpretations by those who were present during the time of the maternal and newborn illness or death. These illness narratives were taken with the aim of gaining the perspective of those who were interviewed, while giving them a voice and granting them the dignity of being heard. A total of 16 cases, including five women with suspected PPH, two maternal deaths, five newborns with severe illness, and four newborn deaths within the last 6 months prior to the study were included. Participants were asked to describe their ideas and experiences regarding the process of recognition of illness, decision-making, and the care-seeking pattern for maternal and newborn complications. Interviews were conducted within ten sub-districts and 15 villages of the Jayawijaya district during a period of 2 months in 2015. Most of the interviews were conducted in the local language.

The research team included two academically trained qualitative researchers who spoke Bahasa Indonesia, two research assistants, four field staff, and three translators who spoke the local language. We trained together prior to data collection in role-plays, practice interviews and discussions on maternal and newborn complications. The interview guides had been field-tested in Jayawijaya and revised before being used for the actual interviews.

A time-by-event matrix instrument was developed to facilitate the recording of the illness narratives. The instrument depicted the recording of textual responses by specific group members that detailed the circumstances surrounding the event and any factors associated with the identification of and response to the event. The matrix included a record of decision-making processes, preferred types of treatment/care, and the perceived quality of the barriers to seeking care. Informants’ demographic and social characteristics were also captured.

After interviews were completed, the research team developed debriefings. Recordings were transcribed verbatim. Some cases in which local dialects were used were translated into Bahasa Indonesia. Coding of these transcripts was performed based on a codebook developed by the TRAction team. The coding process used Nvivo qualitative analysis software, both in Bahasa Indonesia and English. The produced coding results were based on the type of case (maternal illness, maternal death, neonatal illness, or neonatal death) and were used as a reference to create a matrix based on the determined variables. The debriefings and transcripts were then reviewed by researchers and consultants. These reviewers provided feedback to the research team with instruction to clarify any unclear information, to re-interview selected respondents to gather additional information, and to suggest improvement for future data collection. All final transcripts were translated into English and given a unique identification. Each theme or topic was organized into larger categories with various subthemes, which constituted the foundation of synthesizing and conceptualizing the relationships among the data.

### Ethical consideration

Because the research in this study involved human participants, Institutional Review Board (IRB) clearance was obtained from the Expert Committee on Research and Research Ethics from the University of Indonesia to approve the procedures as ethical. This approval was effective during the research period of this study, for up to a year (April 2015–April 2016). To further the ethical soundness of this study, verbal informed consent was received before the start of every interview.

## Results

### Maternal illness and death cases

#### Recognition of postpartum hemorrhage

Qualitative findings indicate that in the geographical area where information was collected, bleeding was a commonly recognized danger sign if present during pre-labor, and depending upon severity during the post-delivery phase, by women included in the study, as well as family members—husband included—and birth assistants. Knowledge on recognition of postpartum hemorrhage was probably dependent upon previous obstetric delivery experience, since most PPH cases included occurred among multi-parous women. Causes of maternal bleeding were in many cases attributed to cultural factors, such as the belief that past mistakes affect the birthing process, as well as the placenta’s (“baby brother” or *kakka* in local terms) responsibility for bleeding. Bleeding severity was subjectively determined by visual observation of the amount of blood that seeped in during a short period of time. No device was used to standardize description of bleeding among included subjects. A respondent who experienced a PPH stated:

“I was bleeding a lot. It was as a full amount of plastic bag here. Similar to this plastic bag ... One plastic bag of blood, and then I was unconscious. It was three times. Because I was lack of blood.” (Focal women of maternal illness case)

Most of the cases included were not assisted by skilled birth attendants but attended by family members and/or elder women. The cause and symptoms of most PPH cases were attributed to retained placenta (“baby brother” in the local term) combined with a supernatural cause, i.e., past mistakes/wrongdoing by the woman or her husband.

#### Care-seeking for postpartum hemorrhage

The decision to seek care lies predominantly with the husband and the mother’s uncle who also plays a major role in the decision-making process. Typically, the maternal uncle’s suggestions or views are considered more than those of other family members. This happens because in Jayawijaya culture, the maternal uncle has a responsibility to protect his niece. When the severity of danger signs is considered high, the uncle’s approval might be overridden by the husband.

**Fig. 1:**
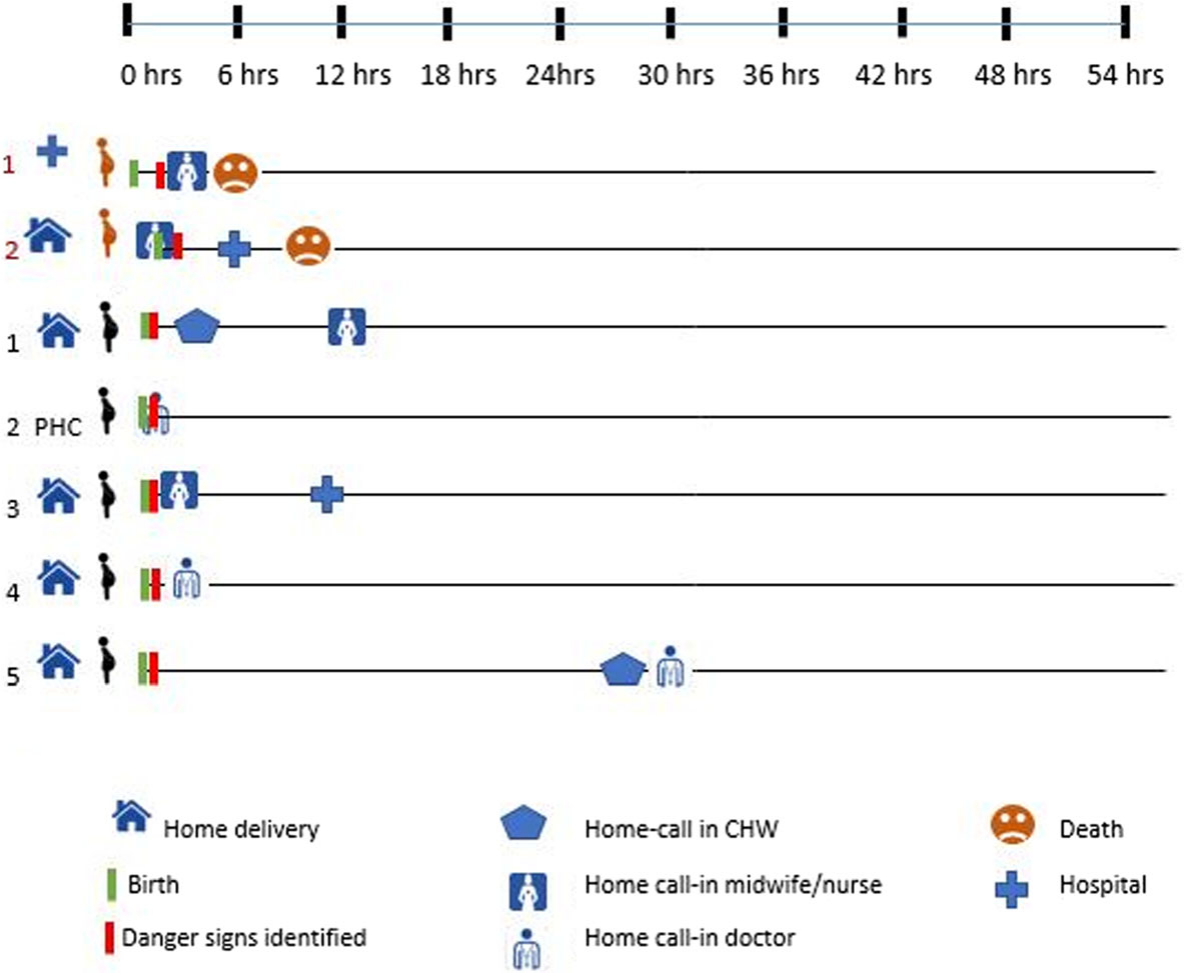
Timeline of maternal cases and patterns of care-seeking and treatment for maternal complications

There was no significant lag time between the decision-making stage and the process of seeking care for the maternal cases. On average, the interval between symptom recognition and the decision to seek care in the home was less than 6 h (Fig. 1). Rapid decision-making usually occurred when visible symptoms of excessive bleeding began to appear. In most of the cases, the husband’s motivation to seek care was influenced by the cultural association of maternal sickness and death with the husband’s wrongdoing. As a consequence, a husband has to pay his wife’s family a compensation for his wrongdoing and lack of care that led to his wife’s death. In some cases, this can also lead to intertribal warfare between the husband’s family and his wife’s family. A respondent whose wife died after delivery even though he already brought her to the hospital stated:

“The decision was from me, because I was afraid. I was in charge in handling the case, I was afraid to be blamed. Therefore, I asked the family of her to take her to the hospital immediately … I did not want to be sued.” (Husband of maternal death case)

Most of the cases received first referral assistance whether it was provided at home or facility within 6–12 h after care sought. The two death cases occurred at the health facility mainly because of delay in identifying the complication and/or receiving appropriate treatment, i.e., non-availability of blood.

#### Care in the home

In Papua, there is preference for home births. The great majority of obstetric deliveries are self-attended by the pregnant woman with or without female communal support (elder women). Only in a limited number of cases does a husband support the delivery process. Once an obstetric complication is recognized, in our study limited to PPH case, the decision to seek care outside the home is done immediately. The first point of seeking care, in the majority of cases, is the local midwife, or the closest health provider (see Fig. 1), who determines the need for referral or home treatment. The decision about provider choice was influenced by the proximity, trust, and close relationship with midwives or doctors.

In the majority of the PPH cases included in the study, uterotonic medication was started at home by the healthcare provider. In most cases, an IV line was established and the first dose of intramuscular oxytocin provided before transportation to the third level of care. In addition, some families perform the ritual of “confession.” The ritual is done by collecting family members and confessing past mistakes. This is done with regard to family beliefs about the cause of symptoms influenced by past mistakes as described in the section on recognition of postpartum hemorrhage.

#### Care outside the home

Maternal care outside the home is provided either at the health center or at the hospital level. Most of the maternal complication cases in our study sought outside care at the hospital level. Poor quality of care for maternal complications was found at EmONC (emergency obstetric and newborn care) services. Both maternal deaths examined suffered from delays in receiving appropriate treatment. Blood transfusion was not readily available in both of the maternal deaths. The first maternal death (see Fig. 1), according to description, was a case of PPH after labor induction (delivery process started at home with referral to the hospital by the midwife), delayed identification of post-delivery complication, and delayed treatment (unavailability of blood). The second maternal case was a home delivery by a midwife with early identification of complication, 1 to 2 h delayed transportation to hospital, and delayed treatment at the hospital due to unavailability of blood for a transfusion.

#### Transfers, referrals, and compliance with referral

All hospital referrals from home were completed. Common barriers for delayed compliance with referral were transport unavailability at nighttime, weather (storms), distance, and cultural living arrangements (husband and wife living in different houses or *honais* within the same compound). In addition to driving factors for seeking care, there are several other factors that also become hindrances. One of the delays in seeking care is the availability of health providers that can be reached at any time. In one case (case number 2, Fig. 1), treatment delay occurred by the absence of a midwife or doctor on standby in the village, resulting in the family having to wait for 2 days until the doctor returned to duty in PHC. If the first treatment/care is done by CHWs or a local midwife and needs to be referred to a hospital, transportation availability factors also affect the time to get further care.

Some barriers occur when the husband and wife stay in different *honais* during the childbirth process, which inhibits the decision to seek care because the husband does not directly know about the condition.

### Newborn illness and death cases

#### Recognition of newborn danger signs

In general, mothers and family members had limitations to recognize newborn danger signs and symptoms. A mother whose baby died stated:

“The baby was carried in noken (traditional bag), two days after the birth, the baby navel suppurated and there were small pustules, and I thought that the pustules were for thickening the baby’s skin, so I left them as they were”.

Perceived severity varies depending on symptom types. For example, moderate vomiting and umbilical/skin infection were perceived as low severity whereas continuous crying and breathing difficulty were considered as high severity. Most symptoms were first recognized by the mother, whereas the father’s involvement in newborn symptom recognition was usually low due to his living in a different *honai* where he could not keep consistent watch over the baby.

From the medical side, factors that were often assumed to be a cause of newborn illness or death were prematurity and improper feeding. Nonmedical factors assumed as a cause included parents’ past mistakes (stealing, cheating, injuring others, violating custom laws, etc.), disturbances caused by demons, conditions of the house environment (e.g., effects of smoke furnaces), procedural errors during delivery (the umbilical cord being too strong or mucus being present in the baby’s mouth during delivery), bad habits of the family (e.g., smoking near the baby), and the feeding of foods other than breast milk before the age of 6 months (such as *hipere*/sweet potato starting from month two or three after birth).

#### Care-seeking

Most of the decisions to seek or not seek care for newborns involved the mother as the main decision-maker. In some cases, the mother and father made decisions together and in some cases also involved the mother’s brother. The role of the mother’s brother in important household decision-making is significant in this region. The pattern of seeking care for cases of newborn illness and death usually began with home treatment, either performed solely by the family or by caregivers brought in from outside the house (religious leaders, CHWs/midwives/doctors of the primary health center). The belief that a child’s sickness results from a parent’s wrongdoing and black magic might delay the decision to seek care. First, when parents begin to recognize danger signs, the family joins together to make a mutual confession of mistakes. This confession also may be accompanied by prayer performed by family, neighbors, or pastors. Once confession has been made, the baby is believed to recover from his/her illness. However, in some instances in which the parents have made mistakes, such problems must be resolved by paying a penalty to the aggrieved person or parties. For example, if the husband previously had an affair with another woman, the husband’s family must pay a fine of pigs to the wife’s family. The belief presented here is that if these problems are solved, the baby’s condition will improve. A father of a newborn illness case mentioned:

“We also did confession, who knows that probably I did something bad and wrong to other people, that was probably what made my baby sick.”

**Fig. 2:**
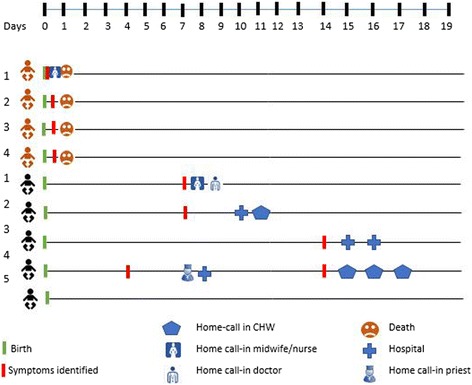
Timeline of newborn cases and patterns of care-seeking and treatment for newborn complications

If the baby’s condition did not improve, health workers would then recommend treatment from better health facilities, such as the hospital (Fig. 2).

#### Care in the home

Home treatments for sick babies consisted of methods such as massaging the baby’s back, praying, wearing objects that are considered to have curing abilities (like a bracelet of threads), and the sprinkling of salt around the house to eliminate interference by demons. In cases where families chose to seek help from health professionals, the families first contacted health personnel who had a relationship with the baby’s family. Only after the health workers could not handle the case in the home was the baby brought to the hospital.

For half of the cases in this study, families chose not to seek help from a healthcare provider (see Fig. 2). This could be the result of several factors mentioned within the interviews, such as the mother’s lack of knowledge of danger signs, the relinquishment of the condition to God, financial constraints, and the location of health facilities. For instance, in one case of newborn illness, the parents did not seek treatment from a health facility because of the lack of adequate finances for transportation to the clinic. Instead, the mother chose to surrender the condition of the baby to God.

#### Care outside of the home

When healthcare workers could not handle the case within the home, the parents were advised to bring the baby to a health facility, such as a clinic or hospital. However, the study showed that care outside of the home was sought only in a few cases of newborn illness, and almost always after 48 h of illness recognition (see Fig. 2), and mostly at hospital level. Primary care facilities were not mentioned during the study as levels of care where families would seek medical help during newborn illness. All four newborn deaths included in the study occurred within 24 h after birth.

#### Transfers, referrals, and compliance with referrals

After implementation of the National Social Insurance (JKN) program in 2014, the health referral system in Indonesia, including Jayawijaya, is using a cascading referral pathway [31]. For non-emergency cases, the patient has to seek care at a first-level health facility (i.e., the local health center). If the healthcare workers of the health center cannot handle such patients, they are then referred to a more adequate health facility, such as the hospital. For emergency cases, patients can bypass the primary health center and go directly to the referral health facilities (e.g., district hospital). For maternal and newborn emergency cases, usually each referred patient is to be accompanied by the healthcare worker who referred them and is to be provided with a referral letter from the health facility that issued the referral. However, public knowledge in Jayawijaya of this referral system seems to be lacking, given that the JKN program just started 1 year before the study. The study found that for newborn complications, most of the cases sought care at a primary health center, whereas for maternal-related complications, referral was directly linked from point of service to hospital. This might reflect that recognition of maternal complications is much better than newborn complications.

Factors that hindered the seeking of treatment, such as referred treatment, included the cultural restriction to bringing a newborn baby outside the home, poor weather conditions, distance to health facilities, bad experiences with healthcare, maternal weakness after childbirth, and transportation costs. As Jayawijaya district has implemented national health insurance that allows citizens to go to government health facilities free of charge, the largest financial factor that has become an obstacle is the cost of public transportation to get to such health facilities (Rp 25,000–30,000 from villages to the city and Rp 5000–10,000 within the city). Other transportation issues consist of the unavailability of public transportation during night hours.

## Discussion

The results of the study highlight differences of recognition and care-seeking behaviors between maternal and newborn complications. To our knowledge, there is no study that explores the differences of recognition and care-seeking of maternal and newborn complications at one study site in Indonesia. Some studies [31] carried out in the field have recorded pregnant women’s and caregivers’ limited knowledge of postpartum excessive bleeding as a danger sign, yet our data suggest that knowledge of such a danger sign was common in this context.

Compared with the maternal cases, this study found mothers’ and caregivers’ recognition of newborn illness at home was poor and/or delayed due to traditional beliefs. One study in Indonesia identified maternal knowledge of neonatal danger signs as the second major risk factor for neonatal death [32], and additional studies have recognized the importance of early recognition for newborn survival [33]. Most illness-related symptoms were ascribed to either prematurity, improper feeding, or cultural-related spiritual beliefs. Based on the care-seeking patterns shown by participant families, the strongest barrier to early skilled care-seeking was not lack of danger sign recognition, but rather cultural beliefs related to ideas of what causes newborn illness. The study by Okafor (2000) in Nepal [9] and by Stewart et al. (1994) in Ghana [34] found very similar issues around care-seeking in the newborn period related to cultural beliefs, such as the need to “watch and wait” in Nepal, and “not for hospital” in Ghana. Hill et al. (2008) also found similar characteristics to the ascription of cultural (evil forces) to newborn disease with consequential preference for traditional treatment [35].

The main figure in the decision-making power related to newborn illness was the mother, supported by her husband, whereas for maternal cases, the key decision-making power was the husband. In both cases, maternal brothers were also involved during the decision-making process. In designing interventions for the purpose of increasing service demand to decrease maternal and newborn mortality, perhaps the target audience will be different considering the different decision-makers of maternal and newborn cases. Few studies have documented the person who decides when and where to seek care once newborn illness has been identified. A study in rural Ghana by Thadeus et al. (2004) [36] found that several members were involved in the care-seeking decision process, whereas husbands were key in financial decision-making.

The great majority of included cases, regardless of danger sign and/or associated severity, started with home care and treatment, either performed by family members or by community-based or health professional caregivers. The first step included home remedies (like praying and sprinkling of salt) to address cultural-related causes, followed by calling in known health workers in cases of absent improvement. Despite the existence of a government referral system, most cases, regardless of the final outcome, stayed at this level, never reaching care outside the home. We found in this region of Indonesia a clear inclination for home treatment (mostly influenced by traditional practices, reinforced by systemic barriers), albeit higher household and community investment for maternal care-seeking outside the home, upon perceived health complication. The preference for home treatment for newborn disease has been reported by multiple studies in Ghana [37], Bangladesh [38], as well as for children under 5 years of age in Ethiopia [39], and Egypt [40]. Most barriers found for seeking care outside the home were related to either a sense of fatalism and/or lack of finances for transportation. In those cases, where healthcare was sought outside the home, on average, this took place between 6 to 12 h in case of maternal illness and 4 days after the onset of the newborn’s illness and always involved bypassing primary care, choosing hospital care as the primary option. As mentioned before, several studies in other countries share similar delays in newborn care-seeking [9], most of them due to a common preference to have the newborn treated at home and by traditional healers. Bypassing more peripheral health clinics in preference for a hospital setting differs from other studies, where Mesko’s points, the higher the level of health worker, the more likely he or she will be seen as a stranger, a social superior, and one whose otherness is magnified by differences in language, ethnicity, and caste.

For both maternal and newborn cases, the similarities are the concept of wrongdoing (or “sin” in the Christian vocabulary) as the cause of sicknesses. Therefore, verbal confessions or compensation payments have to be pursued to heal the sickness first. This similar cultural belief was also documented in Papua New Guinea [41]. The involvement of traditional leaders, e.g., religious and spiritual leaders, therefore becomes critical in improving care-seeking for maternal and newborn complications in this region.

### Limitations

As with any qualitative study, inference of evidence outside the specific area of data collection is limited; therefore, caution needs to be included when applying beyond the study site. One limitation to this study was that case identification and subsequent geographical location for many of the participants was challenging. Typically, patients who came from remote sub-districts or other districts used the address of relatives who lived around the nearest health facilities. However, these patients would usually return to their own homes immediately after treatment was completed, causing difficulty in locating them. In addition, because the recording of addresses in the data register was not complete, finding the location of many cases proved difficult. A second limitation to the study was the cultural norm of Jayawijaya requiring the husband’s presence when interviews were conducted with the wife or family. Because of this, some interviews were delayed, while others could not be conducted at all, due to the husband’s limited time and geographical factors. Other problems included language barriers between interviewers and interviewees, as well as the translators often interpreting and concluding answers of the informant. As usual with qualitative studies, the quality of information is highly dependent on individual’s skills of investigator; moreover, recall bias limits inference of information.

## Conclusions

Our first conclusion points to the differences in recognition of danger signs and care-seeking behaviors between maternal and newborn cases in this study sample. Efforts directed toward household and community recognition of danger signs should prioritize newborns. Redesigning established community platforms, such as the midwifery program, to strengthen the newborn component might be a place to start. For maternal health, emphasis needs to be placed on supply side solutions, and for newborn health, emphasis needs to be placed on demand and supply side solutions, probably including community-based interventions. Bringing newborn health services closer to communities by improving the role community health workers play in newborn health as well as improving the capacity to deal with newborn health at the *puskesmas* level are important interventions for improving the supply side. Secondly, there is importance and need of contextualized information for the design of programs aimed to affect maternal and newborn health. The information collected needs to be integrated within the design of behavioral change programs in rural setting of Papua. And lastly, given the prevailing cultural norms in this area of Indonesia, the role of faith leaders should be explored in changing social norms in aid of newborn health.

## Abbreviations

CHW: Community health worker
EmONC: Emergency obstetric and newborn care
IRB: Institutional Review Board
MMR: Maternal mortality ratio
NMR: Neonatal mortality ratio
PHC: Primary health center
PMNCH: WHO Partnership for Maternal, Newborn and Child Health
PPH: Postpartum hemorrhage
TRACtion: USAID’s Translating Research into Action
